# From J. Edw. Line

**Published:** 1884-04

**Authors:** J. Edw. Line

**Affiliations:** Rochester, N. Y.


*Editor Independent Practitioner:*

Rochester, N. Y., March 21, 1884.

*Editor Independent Practitioner:*

I find on page 152, of your March number, what purports to be a report of my remarks at the late union meeting of the Seventh and Eighth District Dental Societies, wherein I am made to say of the National Board of Examiners, "there are good men in it, and there are bad men in it," etc. I said nothing of the kind; but if your readers will substitute "Association" for "Board," make "good" to mean the fair-play element of the association, and "bad" that element whose motives are open to question, or whose knowledge of New York's affairs is nothing more than garnished ignorance, I will let it stand as published, without further request for change. Respectfully,

J. Edw. Line.

